# Generalized Linear Mixed Models for Binary Data: Are Matching Results from Penalized Quasi-Likelihood and Numerical Integration Less Biased?

**DOI:** 10.1371/journal.pone.0084601

**Published:** 2014-01-09

**Authors:** Andrea Benedetti, Robert Platt, Juli Atherton

**Affiliations:** 1 Department of Medicine, McGill University, Montreal, Canada; 2 Department of Epidemiology, Biostatistics & Occupational Health, McGill University, Montreal, Canada; 3 Respiratory Epidemiology and Clinical Research Unit, Montreal Chest Institute, Montreal, Canada; 4 Department of Pediatrics, McGill University, Montreal, Canada; 5 Département de Mathématiques, Université de Québec à Montréal, Montreal, Canada; Arizona State University, United States of America

## Abstract

**Background:**

Over time, adaptive Gaussian Hermite quadrature (QUAD) has become the preferred method for estimating generalized linear mixed models with binary outcomes. However, penalized quasi-likelihood (PQL) is still used frequently. In this work, we systematically evaluated whether matching results from PQL and QUAD indicate less bias in estimated regression coefficients and variance parameters via simulation.

**Methods:**

We performed a simulation study in which we varied the size of the data set, probability of the outcome, variance of the random effect, number of clusters and number of subjects per cluster, etc. We estimated bias in the regression coefficients, odds ratios and variance parameters as estimated via PQL and QUAD. We ascertained if similarity of estimated regression coefficients, odds ratios and variance parameters predicted less bias.

**Results:**

Overall, we found that the absolute percent bias of the odds ratio estimated via PQL or QUAD increased as the PQL- and QUAD-estimated odds ratios became more discrepant, though results varied markedly depending on the characteristics of the dataset

**Conclusions:**

Given how markedly results varied depending on data set characteristics, specifying a rule above which indicated biased results proved impossible.

This work suggests that comparing results from generalized linear mixed models estimated via PQL and QUAD is a worthwhile exercise for regression coefficients and variance components obtained via QUAD, in situations where PQL is known to give reasonable results.

## Introduction

Increasingly, data are collected in which the standard assumption of independence between observations is not met. This could include data that consist of multiple observations on a subject over time or subjects who are clustered in some way (e.g. classes within schools, or households within neighbourhoods). As computational power has grown, analytic methods have been extended to handle increasingly complex data structures.

If the association between observations on the same cluster/subject is not accounted for in the analytic strategy, inference associated with the estimated parameters may not be correct [1]. When the outcome is binary, one of the main analytic approaches in this context are generalized linear mixed models (GLMMs) [2].

GLMMs extend the linear mixed model to deal with outcomes with non-normal distributions. In particular, GLMMs can handle binary outcomes. In GLMMs, subject-specific random effects, usually normally-distributed, are incorporated in the model. In this way, the second order structure or correlation between subjects in the same cluster can be described and accounted for. When the outcome is binary, in GLMMs the regression coefficient is estimated conditional on the random effect [2], and as such, has a subject-specific interpretation [3], [4].

To estimate the parameters in the GLMM, maximizing the exact likelihood involves an intractable integration. Several approaches have been proposed to get around this. A commonly used method is penalized quasi-likelihood (PQL), proposed by Breslow and Clayton [5]. In this implementation, a Laplace approximation is used, resulting in an approximated likelihood function with Normal distribution [5]. One advantage of PQL is that it can accommodate complex correlation structures. However, estimates can be badly biased especially with few subjects per cluster, low event rates, or high inter-cluster variability, because the method uses an approximation to the likelihood [6]–[8].

The main competitor to PQL is numerical integration via adaptive Gaussian Hermite quadrature (QUAD) [9], [10]. While QUAD is not computationally efficient for multidimensional random effects (e.g. time series), it can perform adequately with few random effects [1]. While quadrature provides accurate estimates for regression coefficients under a variety of conditions, convergence of QUAD is often a problem, particularly when variance parameters are close to zero or cluster sizes are small [11], [12].

Despite many studies investigating the statistical properties of parameter estimates from generalized linear mixed models (GLMMs), it still remains somewhat unclear under what conditions good properties can be expected from either of these methods. In particular, when the number of clusters is small and the number of subjects per cluster is small, neither PQL nor QUAD are guaranteed to give good results for regression coefficients [13]. Similarly, estimated variance components are often biased with both approaches (e.g. [11]).

If matching results from GLMMs estimated via PQL and QUAD indicated relatively unbiased regression coefficients or variance parameters, this could be an easy “diagnostic” to perform. Both estimation methods are available in SAS and R.

In this work, we investigate systematically whether matching regression coefficients, odds ratios or variance components from PQL and QUAD suggest minimal bias in those same parameters. Moreover, we attempt to develop useable guidelines based on comparing the results from PQL and QUAD. For example, should the comparison be between estimated regression coefficients, estimated variance components or both? Moreover, how close is close enough?

## Materials and Methods

Statistical simulation was used to assess whether matching results from PQL and QUAD indicate less bias.

### Data generation

Our data generation algorithm was developed to generate clustered data. We imagined working in the clustered data context (e.g. children grouped in classes, or people clustered in neighbourhoods), rather than longitudinal, repeated measures type data We generated an outcome (Y_ij_) and a predictor (X_ij_). Here, i denotes the cluster, and j denotes the subject within the cluster. Thus Y_ij_ is the outcome observed for subject j from cluster i. The dichotomous independent variable, X_ij_, was generated from a Bernoulli distribution with probability = 0.5. To generate the corresponding dichotomous outcome variable Y_ij_, first the probability of the outcome was generated from the following logistic regression model:
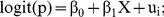
(1)where u_i_ was a random effect generated from a normal distribution with mean = 0 and variance = 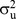. By including u_i_ in the data generation step, correlation between observations in the same cluster is induced. Then the dichotomous Y_ij_ variable was generated from a Bernouilli distribution with the probability of the outcome provided by the logistic regression (equation 1). The number of clusters, number of subjects per cluster, β_1_, variance of the random effect, and proportion of subjects with the outcome were all varied, with levels described in Table 1. For each distinct combination (n = 424) of parameters (“simulation scenarios”), 250 data sets were generated, which gave us adequately precise results, while allowing us to investigate a wide range of simulation scenarios.

**Table 1:** Parameters used for data generation.

Variable	Values taken on
Total number of subjects	Small (150), Medium (450), Large (1500)
Number of clusters (number of subjects per cluster)	6 (25), 15 (10), 30 (5), 75 (2)
	6 (75), 45 (10), 75 (6), 225 (2)
	6 (250), 75 (20), 150 (10), 300 (5), 750 (2)
β_1_	ln(1), ln(1.5), ln(2)
Standard deviation of the random effect (σ_u_)	0, 0.5, 1, 2, 4
Proportion of subjects with the outcome	0.05, 0.2, 0.5

### Data analysis

Two GLMMs (random effects logistic regression models) were fit to the data, including the exposure as an independent variable, and allowing the intercept to vary across the clusters. The model parameters were estimated using penalized quasi-likelihood (PQL) and adaptive Gaussian Hermite quadrature (QUAD). Both models were fit using the GLIMMIX procedure in SAS version 9.2.

### Measures of performance

We collected information on bias and variability of the estimated regression coefficient for X (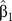), and odds ratio (exp(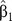)), as well as the estimated variance of the random intercepts (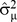), as estimated via PQL or QUAD.

We quantified the proximity of the PQL and QUAD results as the absolute percent difference between the estimated odds ratios, according to the following formula:
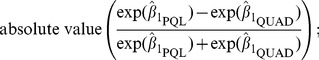
(1)where 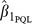 and 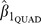 were the regression coefficients as estimated via PQL or QUAD, respectively.

The estimated variance components were compared according to the following formula:
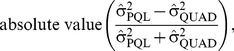
(2)where 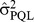 and 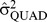 were the variance of the random effects as estimated by PQL or QUAD, respectively.

We also quantified how close results were to the truth, via the following formulae:
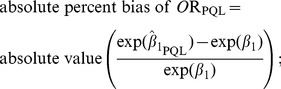
(3)

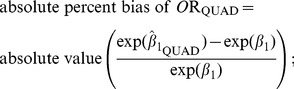
(4)

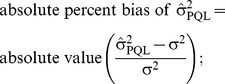
(5)

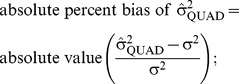
(6)defined as above. When σ^2^ or β_1_ was zero we divided by 1 in formulae 5 and 6.

### Data analysis of simulation results

We removed observations where PQL or QUAD did not converge. Model convergence was defined as a model that produced estimates for relevant parameters and did not return a warning. We estimated convergence for each estimation procedure as the number of simulation repetitions that did converge divided by the total attempted (n = 250).

We estimated the median absolute percent bias of the regression coefficients and random intercept variances as estimated via PQL or QUAD for each simulation scenario.

We fit linear regressions, with absolute percent bias of the estimated odds ratios and absolute percent bias of the variance component (e.g. formulae 3–6) as the outcome and measures of how close PQL and QUAD results were (e.g. formulas 1–2) as the predictors, overall and separately for each combination of data generation parameters (i.e. in 424 distinct data generation scenarios). We report the median estimated slope and interquartile range of the slope, the proportion of scenarios in which the predictor was statistically significant and the median R^2^ and interquartile range of the R^2^ for the models overall (i.e. across all 424 scenarios investigated), as well as by data generation parameters.

Finally, we used mixed quantile regression [14] with absolute percent bias of the estimated odds ratios and variance components (e.g. formulae 3–6) as the outcome and measures of how close PQL and QUAD results were (e.g. formulae 1 and 2) as the predictor of primary interest, and data generation parameters as covariates in the model (i.e. proportion with the outcome, data set size, data set composition, β_1_, σ_u_^2^.) Data set composition categorized data sets as having few large clusters (when the number of clusters was 6), many small clusters (when cluster size was 2), or moderate (all other possibilities). All covariates were entered as dummy variables in the model. Intercepts and the coefficient for similarity of PQL and QUAD results were allowed to vary across simulation scenario.

All data generation and analyses were carried out using SAS/STAT version 9.2 [15], with the exception of the linear mixed quantile regression which was performed in R version 2.14.2 [16].

## Results

Tables 2 and 3 present the median and interquartile range of the absolute percent bias and mean squared error (MSE) of the regression coefficient and variance of the random intercept, respectively, as estimated via QUAD and PQL. Overall, median bias in the PQL or QUAD-estimated regression coefficient was around 30% and increased as the variance of the random effect increased, the proportion with the outcome decreased, the number of observations in the dataset decreased. (See Table 2.)

**Table 2:** Median (Interquartile range (IQR)) absolute percent bias^a^ and mean squared error (MSE) for the regression coefficient as estimated via QUAD or PQL, overall and by data generation parameters.

Data			QUAD		PQL	
Generation Parameter	Value	N^b^	Absolute percent bias β_1_	MSE for β_1_	Absolute percent bias β_1_	MSE for β_1_
			Median (IQR)	Median (IQR)	Median (IQR)	Median (IQR)
**Overall**	**–**	424	0.32 (0.19, 0.54)	0.10 (0.04, 0.28)	0.30 (0.18, 0.49)	0.08 (0.04, 0.20)
**β_1_**	**Ln(1)**	142	0.20 (0.12, 0.32)	0.09 (0.04, 0.26)	0.18 (0.10, 0.28)	0.07 (0.03, 0.18)
	**Ln(1.5)**	141	0.52 (0.32, 0.80)	0.11 (0.04, 0.29)	0.47 (0.32, 0.70)	0.07 (0.03, 0.20)
	**Ln(2)**	141	0.31 (0.19, 0.47)	0.10 (0.04, 0.30)	0.31 (0.20, 0.45)	0.09 (0.04, 0.22)
**σ^2^_u_**	**0**	78	0.21 (0.13, 0.34)	0.04 (0.02, 0.11)	0.20 (0.13, 0.33)	0.04 (0.02, 0.11)
	**1**	117	0.26 (0.18, 0.47)	0.07 (0.02, 0.21)	0.24 (0.16, 0.44)	0.06 (0.02, 0.19)
	**4**	117	0.32 (0.20, 0.54)	0.1 0(0.04, 0.32)	0.31 (0.20, 0.47)	0.08 (0.05, 0.22)
	**16**	112	0.45 (0.28, 0.76)	0.22 (0.07, 1.42)	0.40 (0.24, 0.57)	0.12 (0.06, 0.28)
**p**	**0.05**	112	0.56 (0.32, 0.86)	0.34 (0.1, 30.44)	0.48 (0.30, 0.70)	0.23 (0.08, 0.53)
	**0.2**	156	0.28 (0.17, 0.45)	0.07 (0.03, 0.21)	0.27 (0.16, 0.41)	0.06 (0.02, 0.18)
	**0.5**	156	0.23 (0.15, 0.38)	0.05 (0.02, 0.14)	0.23 (0.14, 0.36)	0.05 (0.02, 0.12)
**Total n**	**150**	130	0.59 (0.41, 0.87)	0.34 (0.19, 17.2)	0.52 (0.35, 0.70)	0.22 (0.16, 0.52)
	**450**	130	0.33 (0.21, 0.49)	0.10 (0.06, 0.25)	0.31 (0.20, 0.44)	0.07 (0.05, 0.20)
	**1500**	164	0.18 (0.12, 0.26)	0.03 (0.02, 0.06)	0.19 (0.11, 0.27)	0.02 (0.02, 0.06)
**Total n**	**150 (6)**	32	0.59 (0.38, 0.87)	0.38 (0.19, 26.96)	0.55 (0.37, 0.77)	0.29 (0.19, 0.55)
**(n cluster)**	**150 (15)**	33	0.57 (0.39, 0.80)	0.30 (0.18, 13.6)	0.52 (0.36, 0.66)	0.24 (0.18, 0.53)
	**150 (30)**	32	0.54 (0.41, 0.76)	0.32 (0.19, 10.61)	0.49 (0.34, 0.68)	0.19 (0.16, 0.42)
	**150 (75)**	33	0.64 (0.43, 0.96)	0.42 (0.21, 70.82)	0.55 (0.35, 0.70)	0.18 (0.14, 0.52)
	**450 (6)**	31	0.32 (0.21, 0.49)	0.09 (0.05, 1.52)	0.32 (0.20, 0.44)	0.07 (0.05, 0.25)
	**450 (45)**	33	0.33 (0.21, 0.46)	0.10 (0.06, 0.23)	0.30 (0.19, 0.44)	0.08 (0.05, 0.21)
	**450 (75)**	33	0.32 (0.23, 0.49)	0.09 (0.06, 0.24)	0.30 (0.19, 0.43)	0.07 (0.05, 0.19)
	**450 (225)**	33	0.34 (0.24, 0.57)	0.12 (0.07, 0.22)	0.32 (0.18, 0.47)	0.06 (0.05, 0.19)
	**1500 (6)**	33	0.18 (0.12, 0.26)	0.03 (0.02, 0.08)	0.18 (0.12, 0.24)	0.03 (0.02, 0.07)
	**1500 (75)**	33	0.18 (0.11, 0.24)	0.03 (0.02, 0.06)	0.17 (0.11, 0.23)	0.02 (0.02, 0.06)
	**1500 (150)**	33	0.18 (0.12, 0.25)	0.02 (0.02, 0.06)	0.16 (0.11, 0.23)	0.02 (0.02, 0.05)
	**1500 (300)**	33	0.17 (0.12, 0.26)	0.03 (0.02, 0.06)	0.20 (0.10, 0.29)	0.02 (0.02, 0.06)
	**1500 (750)**	32	0.18 (0.13, 0.26)	0.03 (0.02, 0.06)	0.20 (0.11, 0.34)	0.03 (0.02, 0.06)

^a^ : First median absolute percent bias of β1 was calculated for each simulation scenario, then summarized across scenarios.

^b^ : This is the number of simulation scenarios used to calculate the information.

**Table 3:** Median (Interquartile range) absolute percent bias^a^ and mean squared error σ^2^_u_ as estimated via QUAD or PQL, overall and by data generation parameters.

Data			QUAD		PQL	
Generation Parameter	Value	N^b^	Absolute percent	MSE for σ^2^_u_	Absolute percent bias σ^2^_u_	MSE for σ^2^_u_
			bias σ^2^_u_	Median (IQR)	Median (IQR)	Median (IQR)
			Median (IQR)			
**Overall**	**–**	424	0.29 (0.16, 0.50)	1.80 (0.18, 30.41)	0.48 (0.30, 0.65)	2.80 (0.15, 65.29)
**β_1_**	**Ln(1)**	142	0.30 (0.17, 0.50)	1.89 (0.18, 37.96)	0.48 (0.30, 0.66)	2.90 (0.15, 67.99)
	**Ln(1.5)**	141	0.28 (0.16, 0.50)	1.86 (0.16, 38.03)	0.48 (0.29, 0.64)	2.80 (0.14, 64.56)
	**Ln(2)**	141	0.29 (0.15, 0.49)	1.57 (0.19, 25.39)	0.48 (0.30, 0.64)	2.73 (0.14, 67.02)
**σ^2^_u_**	**0**	78	0.08 (0.03, 0.15)	0.01 (0.00, 0.04)	0.00 (0.00, 0.01)	0.01 (0.00, 0.02)
	**1**	117	0.43 (0.26, 0.53)	0.40 (0.15, 0.74)	0.43 (0.33, 0.59)	0.37 (0.15, 0.47)
	**4**	117	0.37 (0.20, 0.49)	6.09 (1.54, 13.34)	0.52 (0.42, 0.61)	5.87 (3.63, 8.38)
	**16**	112	0.32 (0.20, 0.56)	243.09 (35.03, 4,426.32)	0.71 (0.56, 0.80)	126.92 (87.12, 163.73)
**p**	**0.05**	112	0.56 (0.36, 0.65)	21.86 (1.57, 476.20)	0.62 (0.54, 0.75)	6.63 (0.70, 129.64)
	**0.2**	156	0.24 (0.15, 0.44)	0.74 (0.07, 14.71)	0.45 (0.12, 0.60)	1.10 (0.07, 42.36)
	**0.5**	156	0.20 (0.12, 0.40)	0.57 (0.05, 9.49)	0.41 (0.10, 0.56)	0.85 (0.05, 33.40)
**Total n**	**150**	130	0.47 (0.36, 0.60)	9.39 (0.63, 2,427.47)	0.54 (0.38, 0.71)	3.45 (0.32, 67.02)
	**450**	130	0.28 (0.21, 0.48)	1.90 (0.21, 24.77)	0.48 (0.31, 0.64)	2.80 (0.15, 63.76)
	**1500**	164	0.17 (0.13, 0.30)	0.57 (0.05, 8.18)	0.45 (0.22, 0.61)	1.74 (0.09, 67.05)
**Total n**	**150 (6)**	32	0.53 (0.49, 0.66)	20.66 (0.66, 235.13)	0.51 (0.44, 0.59)	7.32 (0.68, 37.59)
**(n cluster)**	**150 (15)**	33	0.44 (0.38, 0.54)	9.93 (0.51, 1,863.04)	0.44 (0.34, 0.61)	3.00 (0.32, 77.25)
	**150 (30)**	32	0.40 (0.32, 0.51)	5.86 (0.57, 221.70)	0.49 (0.37, 0.68)	3.73 (0.24, 62.24)
	**150 (75)**	33	0.52 (0.39, 0.63)	11.96 (1.26, 9,290.97)	0.73 (0.57, 0.87)	8.54 (0.40, 193.92)
	**450 (6)**	31	0.50 (0.43, 0.58)	7.35 (0.38, 222.81)	0.45 (0.39, 0.52)	5.62 (0.42, 11.29)
	**450 (45)**	33	0.25 (0.21, 0.36)	2.20 (0.14, 32.09)	0.39 (0.27, 0.56)	2.05 (0.11, 80.68)
	**450 (75)**	33	0.23 (0.20, 0.29)	1.46 (0.14, 23.10)	0.43 (0.30, 0.66)	3.09 (0.13, 111.26)
	**450 (225)**	33	0.29 (0.21, 0.51)	1.91 (0.34, 21.04)	0.71 (0.59, 0.87)	8.51 (0.39, 193.48)
	**1500 (6)**	33	0.48 (0.41, 0.53)	6.82 (0.32, 191.89)	0.43 (0.40, 0.47)	6.65 (0.42, 67.05)
	**1500 (75)**	33	0.16 (0.15, 0.22)	0.75 (0.05, 12.88)	0.26 (0.17, 0.47)	1.11 (0.05, 55.51)
	**1500 (150)**	33	0.14 (0.12, 0.22)	0.51 (0.04, 7.83)	0.33 (0.22, 0.57)	1.74 (0.06, 85.49)
	**1500 (300)**	33	0.15 (0.11, 0.20)	0.40 (0.05, 6.51)	0.47 (0.33, 0.70)	3.62 (0.13, 126.00)
	**1500 (750)**	32	0.16 (0.14, 0.25)	0.57 (0.09, 7.67)	0.72 (0.60, 0.87)	8.25 (0.38, 193.62)

^a^ : Median absolute percent bias of σ^2^_u_ was calculated for each simulation scenario, then summarized across scenarios.

^b^ : This is the number of simulation scenarios used to calculate the information.

On the other hand, the estimated variance of the random intercept was more biased when estimated via PQL than via QUAD (median absolute percent bias was 29% for QUAD vs. 48% for PQL). For both estimation methods, bias increased as the proportion with the outcome decreased and the size of the dataset decreased. For QUAD, bias decreased as the number of clusters increased, while for PQL the opposite was observed. (See Table 3.)

Nonconvergence occurred more often with QUAD than PQL (mean proportion across all simulation scenarios was 8.8 vs. 2.3), and was especially problematic when the proportion of subjects with the outcome was 5% (mean proportion of nonconvergence was 32% for QUAD, but just 8.2 percent for PQL, data not shown). When QUAD did not converge, but PQL did converge, median bias was higher for the PQL-estimated regression coefficient (median = 0.53 with IQR = 0.33–0.88) and variance of the random effect (median = 0.72, IQR: 0.51–0.87) for the estimated. (See Table S1.)

**Figure 1:**
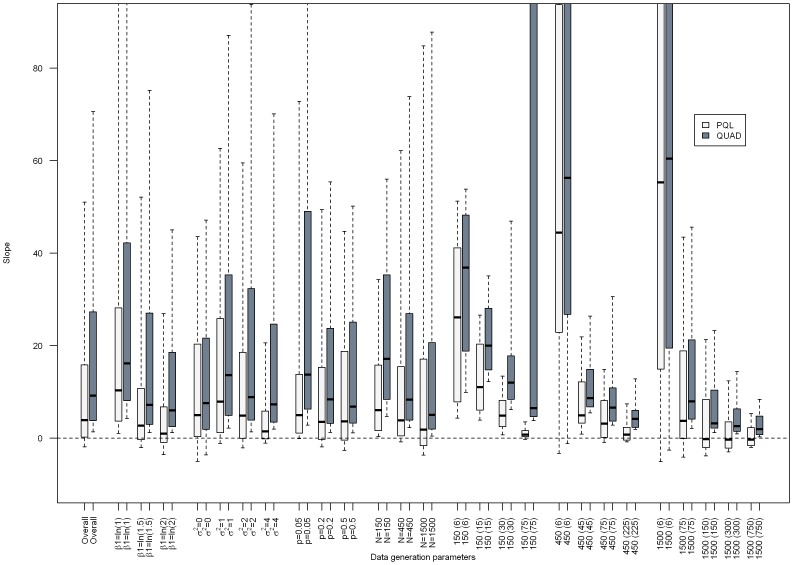
Boxplot depicting the slopes from separate simple linear regressions for the effect of the absolute percent difference in OR_PQL_ and OR_QUAD_ 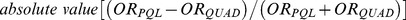 on the absolute percent bias in OR_QUAD_ or OR_PQL_, respectively, overall and by data generation parameters. Median (interquartile range) of the estimated slope is the center of the box, box edges are the 25^th^ and 75^th^ percentile respectively, ends of the dashed lines are the 10^th^ and 90^th^ percentile, respectively.

**Figure 2:**
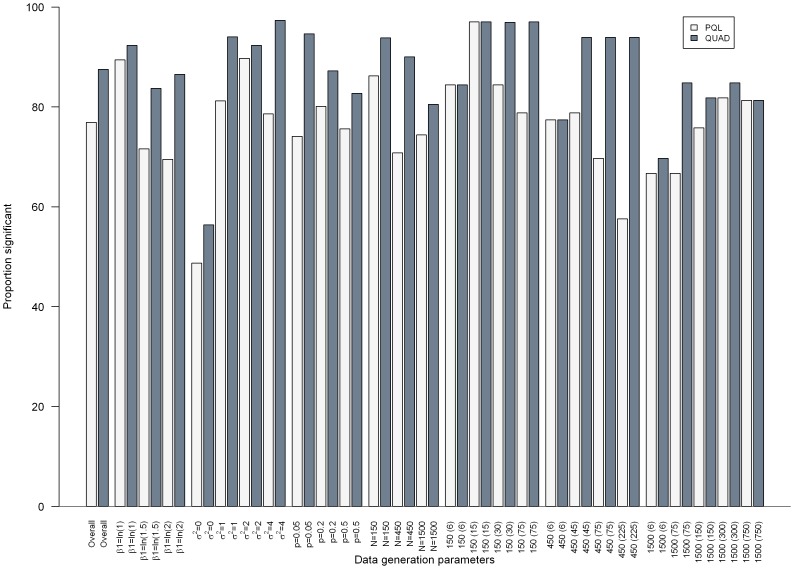
Barplot depicting the proportion of scenarios in which the effect of the absolute percent difference in OR_PQL_ and OR_QUAD_ 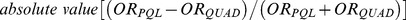 was a statistically significant predictor of the absolute percent bias in OR_QUAD_ or OR_PQL_, respectively from separate simple linear regressions, overall and by data generation parameters.

**Figure 3:**
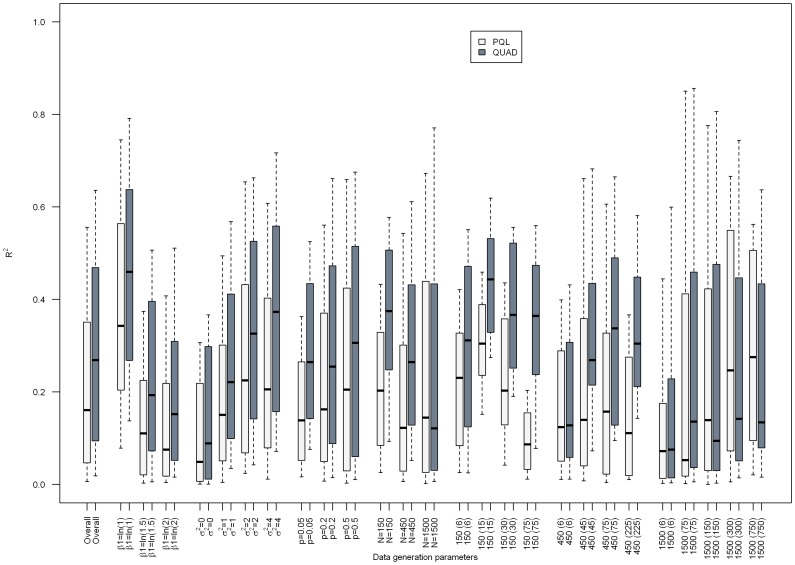
Boxplot depicting the R^2^ from separate simple linear regressions for the effect of the absolute percent difference in OR_PQL_ and OR_QUAD_ 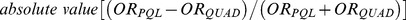 on the absolute percent bias in OR_QUAD_ or OR_PQL_, respectively, overall and by data generation parameters. Median (interquartile range) of the R^2^ is the center of the box, box edges are the 25^th^ and 75^th^ percentile respectively, ends of the dashed lines are the 10^th^ and 90^th^ percentile, respectively.

Figures 1–3 present the results from separate simple linear regressions to model the effect of the absolute percent difference in OR_PQL_ and OR_QUAD_ (equation 1) on the absolute percent bias in OR_QUAD_ (equation 4), and the absolute percent bias in OR_PQL_ (equation 3), respectively, overall and by data generation parameters.

The estimated slope was generally positive when absolute percent bias in OR_QUAD_ was the outcome (See Figure 1). The median slope overall was 8.8, suggesting that for a one percent increase in difference between OR_QUAD_ and OR_PQL_, the absolute percent bias in OR_QUAD_ increased by 8.8. However, the interquartile range was quite wide. For example, the interquartile range of slopes was 7 to 33, 4 to 24 and 2 to 20 for small, medium and large datasets respectively. The estimated slope was never statistically significantly negative. The estimated slope for the effect of absolute percent difference in OR_PQL_ and OR_QUAD_ on the absolute percent bias in OR_PQL_ was statistically significantly negative for 14% (i.e. 60 out of 424) of the data scenarios investigated. The slope was more likely to be negative as the magnitude of β_1_ increased, the proportion of subjects with the outcome increased, the size of the data set increased, if there were few observations per cluster, or the intercluster variability was high. (Data not shown).

Overall, in most simulation scenarios the absolute percent difference in OR_PQL_ and OR_QUAD_ was a statistically significant predictor of the absolute percent bias in OR_PQL_ or OR_QUAD_, respectively, though more often when absolute percent bias in OR_QUAD_ was used as the outcome. (See Figure 2.) The proportion of scenarios in which the absolute percent difference in OR_PQL_ and OR_QUAD_ was a statistically significant predictor decreased as the true regression coefficient increased; and increased as the intercluster variance increased. This proportion decreased as the total number of subjects increased (See Figure 2). The smallest proportion statistically significant were seen when datasets comprised 1500 observations in 6 clusters.

A similar pattern of results was seen for the median R^2^ of the linear regressions (See Figure 3), with results ranging from 0.08 to 0.45, and 0.03 to 0.31 for OR_QUAD_ and OR_PQL_, respectively. The worst results were seen when σ^2^_u_ = 0, while the best results were seen when β_1_ = 0.

We used a linear mixed quantile regression model was used to model the association between absolute percent difference in OR_PQL_ and OR_QUAD_ on the absolute percent bias in OR_PQL_ or OR_QUAD_. We found that overall median absolute percent bias in OR_QUAD_ increased by 6.5% (95% CI: 4.6–8.4) for each 1% difference in the absolute percent difference in OR_PQL_ and OR_QUAD_, after adjusting for data set characteristics. However, this slope was quite variable – the variance of the random effect was 8.2. The association was less strong when absolute percent bias in OR_PQL_ was used as the outcome: median bias in OR_PQL_ increased by 1.2% (95% CI: 0.8–1.6) for each 1% difference in the absolute percent difference in OR_PQL_ and OR_QUAD_, after adjusting for data set characteristics. This slope was less variable – the variance of the random effect was 1.3. See Table 4.

**Table 4:** Results from a linear mixed quantile regression model with absolute percent bias in the odds ratio estimated via PQL or QUAD as the dependent variable and absolute percent difference in the odds ratios as estimated via PQL and QUAD as the independent variable, adjusted for data set characteristics (β_1_, σ^2^_u_, proportion with the outcome (p), total number of observations in the data set and data set composition).

Data		QUAD		PQL	
Generation Parameter	Value	Slope (95% CI)	Var^a^	Slope (95% CI)	Var^a^
**Absolute percent difference in OR_QUAD_ and OR_PQL_**	**–**	6.50 (4.58, 8.43)	8.17	1.17 (0.79, 1.56)	1.28
**β_1_**	**Log(1)**	Ref		Ref	
	**Log(1.5)**	−0.01 (−0.08, 0.06)		0.01 (−0.02, 0.04)	
	**Log(2)**	−0.01 (−0.10, 0.08)		0.00 (−0.05, 0.04)	
**σ^2^_u_**	**0**	Ref		Ref	
	**1**	−0.04 (−0.12, 0.04)		0.00 (−0.04, 0.05)	
	**4**	−0.11 (−0.20, −0.02)		0.00 (−0.03, 0.04)	
	**16**	−0.11 (−0.20, −0.02)		0.02 (−0.01, 0.06)	
**p**	**0.05**	Ref		Ref	
	**0.2**	−0.05 (−0.12, 0.02)		−0.17 (−0.22, −0.11)	
	**0.5**	−0.12 (−0.20, −0.04)		−0.20 (−0.27, −0.14)	
**Total n**	**150**	Ref		Ref	
	**450**	0.00 (−0.07, 0.06)		−0.14 (−0.20, −0.09)	
	**1500**	−0.01 (−0.08, 0.07)		−0.25 (−0.32, −0.17)	
**Dataset composition**	**Many large cluster**	Ref		Ref	
	**Many small clusters**	0.10 (0.09, 0.10)		−0.02 (−0.13, 0.09)	
	**Moderate**	0.29 (0.11, 0.46)		−0.06 (−0.10, −0.03)	

^a^ : This is the variance of the random slope.

In addition to looking at bias in the odds ratios estimate via PQL and QUAD, we also considered using the regression coefficient. However, results were in general, poorer with smaller slopes, lower R^2^ and smaller proportion statistically significant. (Data not shown.)

**Figure 4:**
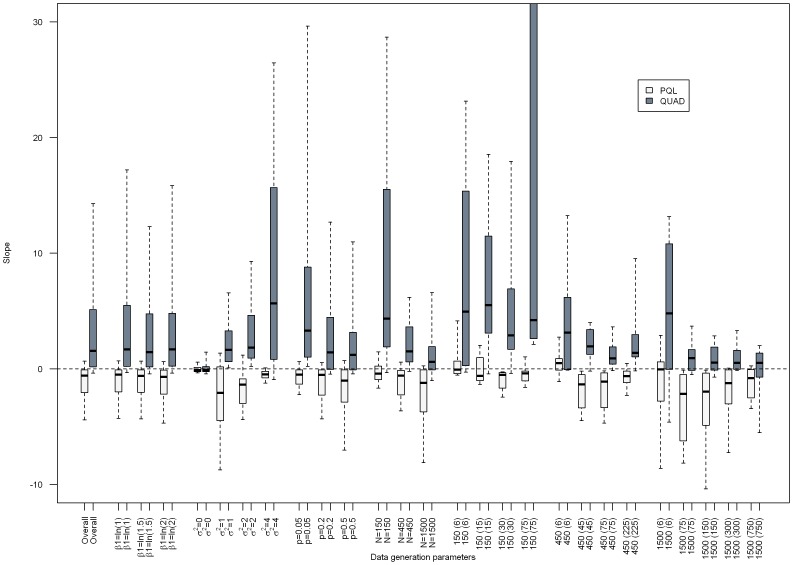
Boxplot depicting the slopes from separate simple linear regressions for the effect of the absolute percent difference in σ_PQL_ and σ_QUAD_ on the absolute percent bias in σ_QUAD_ or σ_PQL_, respectively, overall and by data generation parameters. Median (interquartile range) of the estimated slope is the center of the box, box edges are the 25^th^ and 75^th^ percentile respectively, ends of the dashed lines are the 10^th^ and 90^th^ percentile, respectively.

When absolute percent difference in σ^2^_uPQL_ and σ^2^_uQUAD_ was used as the predictor for the absolute percent bias of σ^2^_uQUAD_ and σ^2^_uPQL_, respectively, the estimated slope varied quite widely, especially when absolute percent bias in σ^2^_uPQL_ was used as the outcome. (See Figure 4.)

**Figure 5:**
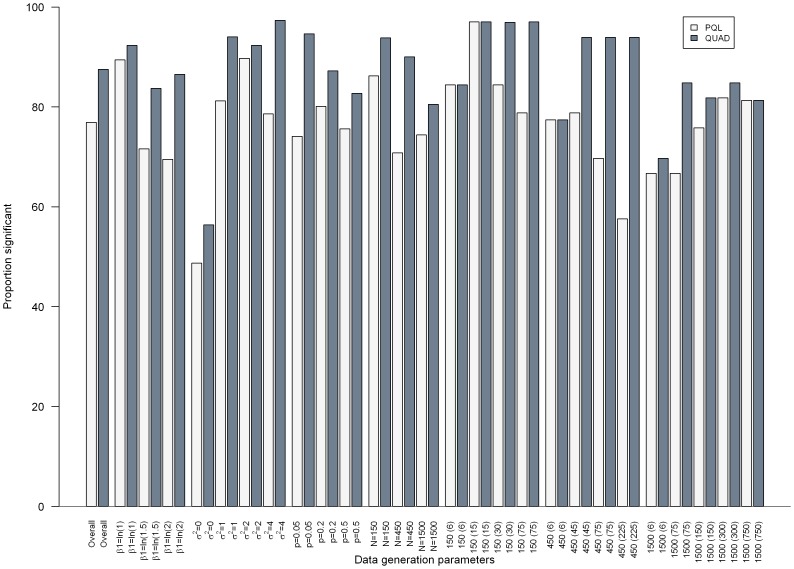
Barplot depicting the proportion of scenarios in which the effect of the absolute percent difference in σ_PQL_ and σ_QUAD_ was a statistically significant predictor on the absolute percent bias in σ_QUAD_ or σ_PQL_, respectively from separate simple linear regressions, overall and by data generation parameters.

**Figure 6:**
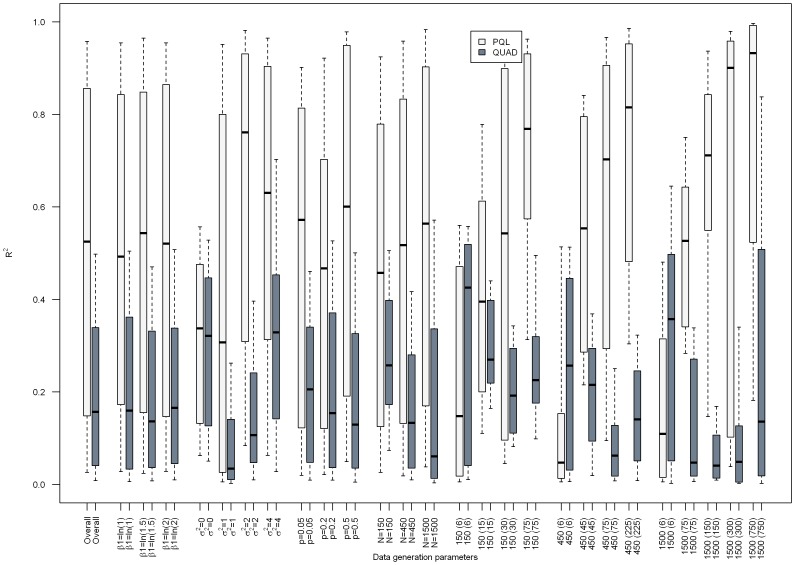
Boxplot depicting the R^2^ from separate simple linear regressions for the effect of the absolute percent difference in σ_PQL_ and σ_QUAD_ on the absolute percent bias in σ_QUAD_ or σ_PQL_, respectively, overall and by data generation parameters. Median (interquartile range) of the R^2^ is the center of the box, box edges are the 25^th^ and 75^th^ percentile respectively, ends of the dashed lines are the 10^th^ and 90^th^ percentile, respectively.

The proportion of scenarios in which this was statistically significant was high (e.g. 85% and 91%, respectively). (See Figure 5.) The median proportion of variance explained by the predictor was 13% and 52%, respectively. (See Figure 6). Indeed, it seemed to be a much stronger predictor for PQL than for QUAD. was the outcome – in that case, the median slope was negative.

The slope was negative in 18% and 75% percent of simulation scenarios for QUAD and PQL, respectively. For PQL, negative slopes were more likely to occur when the variance of the random effect was bigger and when there were fewer subjects per cluster. (Data not shown.)

We used a linear mixed quantile regression model was used to model the association between absolute percent difference in σ^2^_uPQL_ and σ^2^_uQUAD_ on the absolute percent bias in σ^2^_uPQL_ or σ^2^_uQUAD_. The association was not statistically significant for σ^2^_uQUAD_. The association was small and quite variable for σ^2^_uPQL_, after adjusting for data set characteristics. See Table 5.

**Table 5:** Results from a linear mixed quantile regression model with absolute percent bias in σ^2^_u_ estimated via PQL or QUAD as the dependent variable and absolute percent different in σ^2^_u_ as estimated via PQL and QUAD as the independent variable, adjusted for data set characteristics (β_1_, σ^2^_u_, proportion with the outcome (p), total number of observations in the data set and data set composition).

Data		QUAD		PQL	
Generation Parameter	Value	Slope (95% CI)	Var^a^	Slope (95% CI)	Var^a^
**Absolute percent difference in OR_QUAD_ and OR_PQL_**	**–**	−0.01 (−0.21, 0.18)	0.29	0.11 (0.00, 0.22)	1.46
**β_1_**	**Log(1)**	Ref		Ref	
	**Log(1.5)**	0.00 (−0.01, 0.01)		0.00 (−0.03, 0.02)	
	**Log(2)**	−0.01 (−0.02, 0.01)		0.00 (−0.02, 0.02)	
**σ^2^_u_**	**0**	Ref		Ref	
	**1**	0.15 (0.11, 0.19)		0.31 (0.22, 0.40)	
	**4**	0.12 (0.07, 0.18)		0.38 (0.33, 0.42)	
	**16**	0.15 (0.07, 0.23)		0.50 (0.46, 0.53)	
**p**	**0.05**	Ref		Ref	
	**0.2**	−0.10 (−0.13, 0.06)		−0.06 (−0.09, −0.04)	
	**0.5**	−0.12 (−0.16, −0.08)		−0.09 (−0.12, −0.06)	
**Total n**	**150**	Ref		Ref	
	**450**	−0.11 (−0.15, −0.07)		−0.03 (−0.06, −0.01)	
	**1500**	−0.18 (−0.24, −0.13)		−0.06 (−0.12, 0.00)	
**Dataset composition**	**Many large cluster**	Ref		Ref	
	**Many small clusters**	−0.17 (−0.34, 0.00)		0.14 (0.07, 0.21)	
	**Moderate**	−0.15 (−0.22. −0.08)		0.03 (−0.07, 0.13)	

^a^ : This is the variance of the random slope.

The absolute difference in σ^2^_uPQL_ and σ^2^_uQUAD_ was not a very good predictor for the absolute percent bias in OR_QUAD_ or OR_PQL_ – fewer models were statistically significant (e.g. 29% overall for QUAD and 16% overall for PQL), R^2^ was low, and the estimated slope was close to 0. (Data not shown.)

The absolute percent difference in OR_PQL_ and OR_QUAD_ was not a good predictor of the absolute bias of σ^2^_uPQL_ or σ^2^_uQUAD_. It was often statistically significant (e.g. 66% and 83% overall for QUAD and PQL, respectively), though R^2^ was usually less than 0.3. In fact, the median slope across all scenarios was negative for PQL. (Data not shown.)

## Discussion

Over time, adaptive Gaussian Hermite quadrature has become the gold standard for fitting generalized linear mixed models with binary outcomes. However, given the greater flexibility in terms of modelling correlation structures available with penalized quasi-likelihood, and better convergence due to simpler estimation, PQL is still used frequently. Moreover, in some scenarios, neither approach uniformly gives good results. In this work, we systematically evaluated whether matching results from PQL and QUAD indicate less bias in estimated regression coefficients and variance parameters.

Overall, we found that the absolute percent bias of the odds ratio estimated via PQL or QUAD increased as the PQL- and QUAD-estimated odds ratios became more discrepant. While the estimated slope for the association between the absolute percent difference in the PQL- and QUAD-estimated odds ratios and the absolute percent bias of the odds ratio estimated via PQL or QUAD varied markedly depending on the characteristics of the dataset, the association for QUAD was almost always positive. In contrast, when using the absolute bias of the OR estimated via PQL as the outcome, the slope was often negative. In fact, it was negative in scenarios that are known to produce biased results for PQL – namely few subjects per cluster and high intercluster variability [5], [17], [18]. In these cases, the higher the discrepancy between the results, the more biased the PQL estimated odds ratio was.

We found that the absolute difference in σ^2^_uPQL_ and σ^2^_uQUAD_ was not a strong predictor for the absolute bias of σ^2^_uQUAD_ or the odds ratios estimated via PQL or QUAD. Moineddin et al. found that with two level data structures, the variance components were extremely overestimated with small groups and slightly underestimated with moderate group size for GLMM estimated via quadrature [19]. PQL has been found to underestimate the variance components when the denominator is small [7]. We found that absolute percent bias for σ^2^_u_ was greater for PQL than quadrature. For PQL, bias was worse when group size was small while for QUAD bias was worse when the number of groups was small.

Given how markedly results varied depending on data set characteristics, specifying some cutpoints above which indicated biased results proved impossible. For example, when identifying odds ratios estimated via QUAD or PQL that were more than 30% biased and using the discrepancy between QUAD and PQL as the test, the area under the curve of the receiver operator curve was 66% for QUAD and 60% for PQL across all scenarios. Despite this, our results show that discrepant results may indicate increased bias.

One strength of this work was the use of simulations to systematically investigate the robustness of the association between similarity in PLQ and QUAD estimates as predictors of bias PQL- and QUAD- regression coefficients and variance components. This allowed us to investigate the impact of a wide range of data set characteristics on these associations. Indeed, we varied data set size and composition, proportion of subjects with the outcome, magnitude of the effect under study, and inter-cluster variability in over 400 distinct data generation scenarios. Despite this, our scenarios were certainly not exhaustive.

Moreover, we made many simplifying decisions. We considered data sets with only one categorical predictor, only one level of clustering, and only generated data with normally distributed random intercepts, not random slopes, or more complicated correlation structures. Finally, we have only compared two methods, whereas some may also have been interested in comparing Bayesian methods of estimation [20], or other approaches.

This work suggests that comparing results from generalized linear mixed models estimated via PQL and QUAD is a worthwhile exercise for regression coefficients and variance components obtained via QUAD, in situations where PQL is known to give reasonable results. Results were less useful for results obtained via PQL. In both cases, results strongly depended on features of the data set, making it difficult to create a simple-to-implement rule.

## Supporting Information

Table S1 **Median (IQR) proportion of samples in which the model did not converge overall and by data generation parameters.**
(DOC)
