# Supplementary material for: Generalized Linear Mixed Models for Binary Data: Are Matching Results from Penalized Quasi-Likelihood and Numerical Integration Less Biased?
Source: PLoS One. 2014 Jan 9;9(1):e84601. doi: 10.1371/journal.pone.0084601 (PMC3886992; doi:10.1371/journal.pone.0084601)
Supplement: Table S1 — Median (IQR) proportion of samples in which the model did not converge overall and by data generation parameters. (DOC) [file pone.0084601.s001.doc]

Supplemental Results

Table S1: Median (IQR) proportion of samples that did not converge overall and by data generation parameters

| **Data** |  |  | **QUAD** | **QUAD** | **PQL** | **PQL** |
| --- | --- | --- | --- | --- | --- | --- |
| **Generation Parameter** | **Value** | **N[[1]](#footnote-2)** | **Median IQR** | **Mean (SD)** | **Median IQR** | **Mean (SD)** |
| **Overall** | **--** | 429 | 0.0 (0.0, 0.0) | 8.7 (22.8) | 0.0 (0.0, 0.0) | 2.3 (10.6) |
| **β_1_** | **Ln(1)** | 143 | 0.0 (0.0, 0.0) | 8.8 (22.9) | 0.0 (0.0, 0.0) | 2.0 (9.5) |
|  | **Ln(1.5)** | 143 | 0.0 (0.0, 0.4) | 8.8 (23.2) | 0.0 (0.0, 0.0) | 2.8 (12.6) |
|  | **Ln(2)** | 143 | 0.0 (0.0, 0.0) | 8.5 (22.4) | 0.0 (0.0, 0.0) | 2.1 (9.4) |
| **σ^2^_μ_** | **0** | 78 | 0.0 (0.0, 0.0) | 0.0 (0.0) | 0.0 (0.0, 0.0) | 0.0 (0.1) |
|  | **1** | 117 | 0.0 (0.0, 0.0) | 5.5 (18.5) | 0.0 (0.0, 0.0) | 0.7 (2.4) |
|  | **4** | 117 | 0.0 (0.0, 1.6) | 8.0 (21.9) | 0.0 (0.0, 0.0) | 1.5 (4.3) |
|  | **16** | 117 | 0.0 (0.0, 40.0) | 18.4 (30.6) | 0.0 (0.0, 1.2) | 6.2 (19.2) |
| **p** | **0.05** | 117 | 12.7 (0.8, 65.2) | 31.8 (34.2) | 0.8 (0.0, 8.8) | 8.2 (19.1) |
|  | **0.2** | 156 | 0.0 (0.0, 0.0) | 0.1 (0.2) | 0.0 (0.0, 0.0) | 0.1 (0.6) |
|  | **0.5** | 156 | 0.0 (0.0, 0.0) | 0.0 (0.1) | 0.0 (0.0, 0.0) | 0.0 (0.1) |
| **Total n** | **150** | 132 | 0.0 (0.0, 1.4) | 8.4 (19.2) | 0.0 (0.0, 4.7) | 5.0 (14.0) |
|  | **450** | 132 | 0.0 (0.0, 0.0) | 8.9 (23.0) | 0.0 (0.0, 0.0) | 2.0 (12.5) |
|  | **1500** | 165 | 0.0 (0.0, 0.0) | 8.8 (25.2) | 0.0 (0.0, 0.0) | 0.3 (1.8) |
| **Total n** | **150 (6)** | 33 | 0.0 (0.0, 1.2) | 2.7 (6.5) | 0.0 (0.0, 8.4) | 8.6 (19.6) |
| **(n cluster)** | **150 (15)** | 33 | 0.0 (0.0, 0.4) | 4.8 (12.2) | 0.0 (0.0, 4.6) | 4.4 (8.7) |
|  | **150 (30)** | 33 | 0.0 (0.0, 7.2) | 9.2 (20.4) | 0.0 (0.0, 4.1) | 5.3 (17.6) |
|  | **150 (75)** | 33 | 0.0 (0.0, 50.0) | 16.8 (28.1) | 0.0 (0.0, 2.9) | 1.8 (3.2) |
|  | **450 (6)** | 33 | 0.0 (0.0, 0.0) | 1.1 (3.2) | 0.0 (0.0, 0.4) | 7.4 (24.4) |
|  | **450 (45)** | 33 | 0.0 (0.0, 0.0) | 5.6 (15.9) | 0.0 (0.0, 0.0) | 0.1 (0.5) |
|  | **450 (75)** | 33 | 0.0 (0.0, 0.4) | 7.7 (20.3) | 0.0 (0.0, 0.0) | 0.4 (0.9) |
|  | **450 (225)** | 33 | 0.0 (0.0, 60.1) | 21.3 (35.5) | 0.0 (0.0, 0.0) | 0.1 (0.2) |
|  | **1500 (6)** | 33 | 0.0 (0.0, 0.0) | 0.4 (1.5) | 0.0 (0.0, 0.0) | 1.2 (3.7) |
|  | **1500 (75)** | 33 | 0.0 (0.0, 0.0) | 3.9 (12.4) | 0.0 (0.0, 0.0) | 0.0 (0.2) |
|  | **1500 (150)** | 33 | 0.0 (0.0, 0.0) | 6.6 (20.6) | 0.0 (0.0, 0.0) | 0.1 (0.2) |
|  | **1500 (300)** | 33 | 0.0 (0.0, 0.0) | 9.3 (27.3) | 0.0 (0.0, 0.0) | 0.3 (0.8) |
|  | **1500 (750)** | 33 | 0.0 (0.0, 65.2) | 23.7 (39.8) | 0.0 (0.0, 0.0) | 0.0 (0.1) |

1. This is the number of simulation scenarios used to calculate the information. [↑](#footnote-ref-2)
